# Reversible disruption of XPO1-mediated nuclear export inhibits respiratory syncytial virus (RSV) replication

**DOI:** 10.1038/s41598-021-98767-2

**Published:** 2021-09-28

**Authors:** Cynthia Mathew, Sharon Tamir, Ralph A. Tripp, Reena Ghildyal

**Affiliations:** 1 Centre for Research in Therapeutic Solutions, Faculty of Science and Technology, University of Canberra, Canberra, ACT 2617 Australia; 2 Karyopharm Therapeutics, Newton, MA USA; 3 Department of Infectious Diseases, College of Veterinary Medicine, University of Georgia, Athens, GA USA

**Keywords:** Virus-host interactions, Antivirals

## Abstract

Respiratory syncytial virus (RSV) is the primary cause of serious lower respiratory tract disease in infants, young children, the elderly and immunocompromised individuals. Therapy for RSV infections is limited to high risk infants and there are no safe and efficacious vaccines. Matrix (M) protein is a major RSV structural protein with a key role in virus assembly. Interestingly, M is localised to the nucleus early in infection and its export into the cytoplasm by the nuclear exporter, exportin-1 (XPO1) is essential for RSV assembly. We have shown previously that chemical inhibition of XPO1 function results in reduced RSV replication. In this study, we have investigated the anti-RSV efficacy of Selective Inhibitor of Nuclear Export (SINE) compounds, KPT-335 and KPT-185. Our data shows that therapeutic administration of the SINE compounds results in reduced RSV titre in human respiratory epithelial cell culture. Within 24 h of treatment, RSV replication and XPO1 expression was reduced, M protein was partially retained in the nucleus, and cell cycle progression was delayed. Notably, the effect of SINE compounds was reversible within 24 h after their removal. Our data show that reversible inhibition of XPO1 can disrupt RSV replication by affecting downstream pathways regulated by the nuclear exporter.

## Introduction

Respiratory syncytial virus (RSV) is a major cause of lower respiratory tract infections affecting a broad demographic, including neonates, children under the age of five, immunocompromised individuals and the elderly^1–3^. Currently, treatment of RSV-associated bronchiolitis is generally supportive including supplemental oxygenation or ventilators^4^. Very limited therapeutic options are available for high risk infants; examples include ribavirin, a synthetic nucleoside analogue that inhibits viral replication^5^, and Palivizumab (Synagis^®^), a highly potent RSV-neutralizing monoclonal antibody that targets the RSV F protein^6^.

Targeting host factors to disrupt viral infection has gained traction in the past decade because of increased rates of resistance against current antivirals that target viral factors^7^. Viruses subvert a limited set of host factors during infection to facilitate various stages of their lifecycle^8^ and disruption of the function of the host factor indirectly reduces or inhibits viral replication^9,10^.

Modulating the nucleocytoplasmic trafficking system, including importin and exportin proteins, is a mechanism used by many viruses at different stages of replication^8^. We have previously shown that RSV M protein is localised to the nucleus early in infection, being exported to the cytoplasm later to play its central role in RSV assembly; disruption of nuclear export of M protein inhibits RSV assembly and reduces viral titre^11–13^. XPO1 is the sole nuclear exporter for over 200 macromolecules and is essential for cell function and survival and irreversible inhibition of nuclear export is not a viable strategy. On the other hand, selective and reversible disruption of XPO1-mediated nuclear transport is sufficient to interfere with cancer progression and viral replication^8,14,15^.

KPT-335 (Verdinexor) is a novel, oral Selective Inhibitor of Nuclear Export (SINE) compound being evaluated in a variety of viral indications as well as in autoimmune/inflammatory diseases^12,16–18^. KPT-335 was tested in healthy volunteers and was found to be fairly safe and tolerated in clinically relevant doses (ClinicalTrials.gov Identifier: NCT02431364). KPT-185 was designed primarily for in vitro studies, and its orally bioavailable analog, KPT-251 has preclinical efficacy against various haematological and solid cancers in mice models^19^. In this study we show that SINE compounds reduce RSV replication when administered therapeutically and discuss the possible mechanisms. Our data shows that SINE compounds reduce the expression of XPO1, delay cell cycle progression and impact cytokine/interferon expression. The current study extends our previous work which showed that treatment with KPT-335 up to 72 h prior to infection inhibits RSV replication, highlighting its potential as a prophylactic agent against RSV^12^.

## Results

### KPT-185 and KPT-335, selective XPO1/CRM1 inhibitors, inhibit XPO1-mediated export and reversibly reduce XPO1 expression

**Figure 1:**
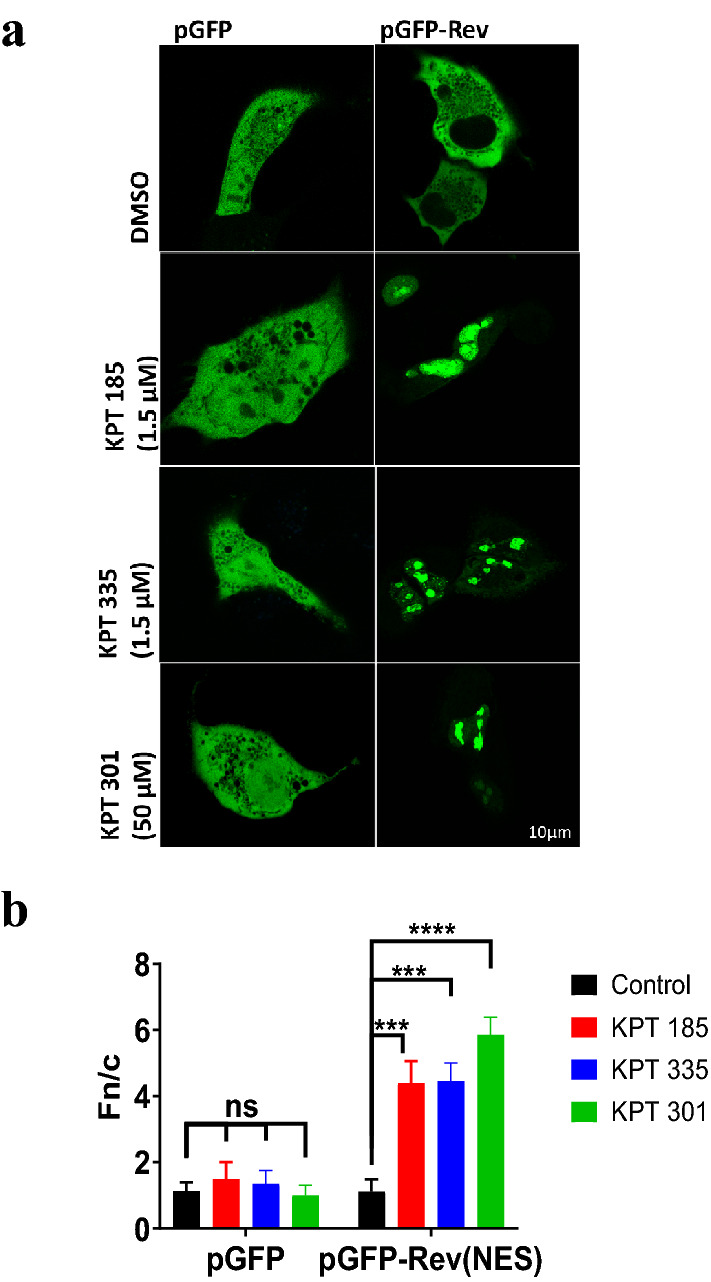
Disruption of XPO1-mediated nuclear export of GFP-Rev (NES) on treatment with SINE compounds. (**a**) A549 cells were transfected with pGFP or pGFP-Rev (NES) using Lipofectamine 2000. 18 h post transfection, cells were treated with SINE compounds or DMSO (control) for 6 h. Live cell images were captured with Nikon Ti-Eclipse confocal system and NIS AR Elements software. (**b**) The subcellular localization of GFP and GFP-Rev (NES) was determined using Fiji ImageJ (vr. 1.52 s) and the equation Fn/c = (Fn − Fb)/(Fc − Fb), where Fn/c is the nuclear/cytoplasmic ratio, Fn is the nuclear fluorescence, Fc is the cytoplasmic fluorescence, and Fb is the background or autofluorescence). Data shown are mean ± SEM, n ≥ 15. Statistical significance was determined using two-way ANOVA with Tukey’s post hoc test with GraphPad Prism v8.4.3. ns: non-significant; ***p < 0.001, ****p < 0.0001. Data is representative of three independent experiments.

HIV-Rev protein uses XPO1-dependent nuclear pathway to mediate the export of viral components into the cytoplasm, and a GFP fused to the NES domain of Rev has been used effectively to study the efficacy of XPO1 inhibitors^13,20^. A549 cells were transfected to express GFP-Rev(NES) or GFP alone (control) and treated with SINE compounds or DMSO for 6 h. GFP alone was present diffused throughout the cell, as expected (Fig. 1a,b), with no significant differences between DMSO- or SINE-treated cells. GFP-Rev (NES) was mostly cytoplasmic (Fn/c of 1.19) in DMSO-treated cells. In contrast, GFP-Rev (NES) localized significantly to the nucleolar and nuclear regions on treatment with 1.5 µM of KPT-185 (Fn/c = 4.39; p = 0.003) or KPT-335 (Fn/c = 4.56; p = 0.003) (Fig. 1a,b) with almost no fluorescence in the cytoplasm. This indicates the effect of SINE compounds on GFP-Rev (NES) was selective to the NES-carrying protein as we have shown previously for KPT-335^12^.

**Figure 2:**
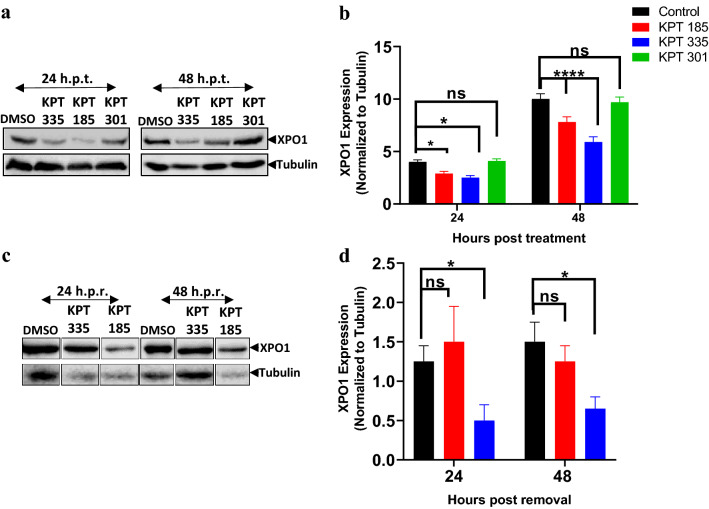
Reduction and recovery of XPO1 expression in cells treated with SINE compounds. (**a**) Uninfected A549 cells were treated with SINE compounds (KPT-335 = 1.5 μM; KPT-185 = 1.5 μM; and KPT-301 = 50 μM) or DMSO for 24 or 48 h. (**c**) Uninfected A549 cells were treated as in (**a**) and further incubated without the compounds for 24 or 48 h. The cell lysates were collected at the respective time points using lysis buffer and analysed using Western blotting. The blot was probed with mouse anti-XPO1 (1:1000; MW = 125 kDa) and rabbit anti-α/β-Tubulin (1:1000; MW = 55 kDa). The bands were detected using Enhanced Chemiluminescence (ECL) kit, Li-Cor Odyssey Fc infrared imaging system and Image Studio Lite software. Please refer to Fig. S1 for full length blots corresponding to the cropped blots presented here. (**b**, **d**) XPO1 protein expression normalized to tubulin was plotted on GraphPad Prism v8.4.3; ns: non-significant; *p < 0.05, ****p < 0.0001. Significance was determined using two-way ANOVA with Tukey’s post hoc test. The images shown are representative of three independent experiments.

XPO1 expression in cell lysates of uninfected A549 cells treated with 1.5 μM KPT-185, 1.5 μM KPT-335, or 50 μM KPT-301 for 24 or 48 h was determined using Western blotting (Fig. 2a). This period would cover two replication cycles of RSV^21,22^. KPT-185 significantly (p = 0.043) reduced XPO1 expression at 24 h post treatment (h.p.t.), and the reduction was bigger at 48 h.p.t (p < 0.0001) (Fig. 2a,b). Treatment with KPT-335 significantly decreased the amount of XPO1 at both 24 h.p.t (p = 0.004) and 48 h.p.t (p < 0.0001). This finding is in agreement with our earlier study^12^ which reported a reduction in XPO1 expression at 24 h.p.i following treatment with KPT-335. No significant difference in XPO1 expression was observed between DMSO-treated and KPT-301-treated cells (Fig. 2a,b). Taken together with its effect on GFP-Rev (NES) localisation, this suggests a different mode of action from that of KPT-185, KPT-335.

XPO1-mediated nuclear export is vital for normal cell functioning^8^. Therefore, it is important to determine if the expression of XPO1 would be restored upon removal of SINE compounds. Uninfected A549 cells were treated with 1.5 μM of KPT-185 or KPT-335 for 48 h, the medium was replaced with tissue culture medium, and cells incubated further for another 24 or 48 h (Fig. 2c,d). XPO1 expression in KPT-185-treated cells recovered within 24 h post removal (h.p.r) to levels similar to DMSO-treated cells. Removal of KPT-335 also resulted in an increase in XPO1 but to a lesser extent and the recovery of XPO1 expression required more time in KPT-335-treated cells. This suggests the effect of KPT-335 treatment lasts longer than KPT-185. Our data agrees with previous literature that treatment with SINE compounds would cause minimal effects on non-infected cells^23^.

### KPT-335 and KPT-185 have high CC_50_ and low IC_50_ values

**Figure 3:**
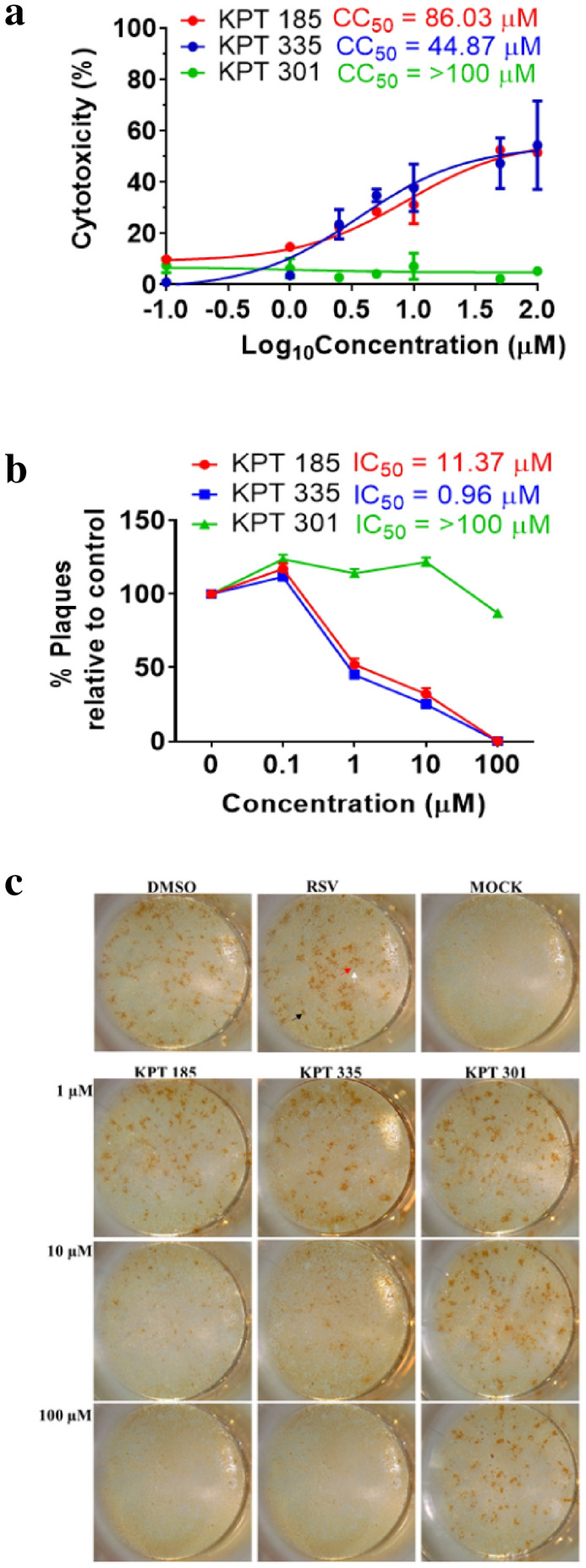
Cytotoxicity and Anti-RSV efficacy of SINE compounds. (**a**) Cytotoxicity of SINEs in A549 cells was determined using Promega CytoTox 96^®^ Non-Radioactive Cytotoxicity Assay. Cells were treated with increasing concentrations of SINE compounds, KPT-185 (red), KPT-335 (blue) and KPT-301 (green), between 0.01 and 100 μM for 48 h. LDH concentrations in the supernatant were analysed as per the manufacturer’s recommendation. Percent cytotoxicity in SINE-treated cells was determined by the LDH concentrations relative to lysed (100% cytotoxicity) cells. Average of three readings for each sample was used to estimate percent cytotoxicity. The values shown are mean ± SEM from three independent experiments. (**b**) Anti-RSV efficacy of SINE compounds was determined using plaque reduction assay. Overnight cultures of cells were infected at MOI = 1 and treated with increasing concentrations of SINE compounds from 2 to 48 h.p.i, fixed and viral titre in the treated and non-treated cells determined using immunoplaque assays. Each sample was analysed in quadruplicate and data shown are representative of three independent experiments. The cytotoxic and inhibitory concentrations (CC_50_ and IC_50_) were determined using non-linear regression analysis in GraphPad Prism v.8.4.3. (**b**) Images of plaques in treated and non-treated wells were taken using Leica EZ24W stereomicroscope and Leica Application Suite software. Comet-shaped plaques (black arrow), medium sized (white arrow) and pinpoint plaques (red arrow) are indicated on the RSV only image. Representative images from three independent experiments are shown. Please refer to Fig. S3 for a magnified image of plaques.

The CC_50_ values for KPT-185 and KPT-335 were 86.03 µM and 44.9 µM, respectively. The percentage cytotoxicity induced by KPT-301 treatment did not exceed 10% even after treatment with the highest dose of 100 µM (Fig. 3a). The anti-RSV efficacy of the SINE compounds was evaluated using immunoplaque assays. A549 cells were infected with RSV for 1 h and treated with increasing doses of SINE compounds from 2 h post infection (h.p.i.) to 48 h.p.i. The percentage reduction in the number of plaques in the SINE-treated A549 cells in comparison to non-treated cells is shown in Fig. 3b. The IC_50_ values for KPT-185 and KPT-335 were 1.3 µM and 0.96 µM at 48 h.p.i, respectively. 100% inhibition of RSV replication was observed following treatment with ≥ 10 µM of KPT-185 and KPT-335. KPT-301 was ineffective against RSV-A2 at all the tested doses (0.1–100 μM), indicating that it is ineffective against RSV.

Viral plaque morphology in human cell lines can gauge infectivity, cytopathic effects, and is used to assess viral fitness^24,25^. The immunoplaque assay used in this study is similar to a quantitative comet assay used to evaluate efficacy of antiviral drugs or antibodies^26,27^. Comet shaped plaques indicate the sample contains infectious particles capable of spreading in contiguous cells^27^. Change in plaque morphology to smaller, blunted comet-shaped plaques or rounder, pin-point plaques indicates presence of fewer infectious particles^27^. Comparison of plaque number and morphology between treated and non-treated samples can provide an indication of efficacy of treatment. Representative images of the plaques in cells treated with SINE compounds from 2 to 48 h.p.i are shown in Fig. 3c. Plaque morphology in KPT-185- and KPT-335-treated cells had fewer comets and scattered pin-point plaques in comparison to DMSO- or KPT-301-treated cells (Fig. 3c). Decrease in viral titre and change in plaque morphology indicate disruption of XPO1 function as a promising post-infection therapy against RSV.

### Treatment with SINE compounds reduced RSV replication with no substantial change in RSV protein expression

**Figure 4:**
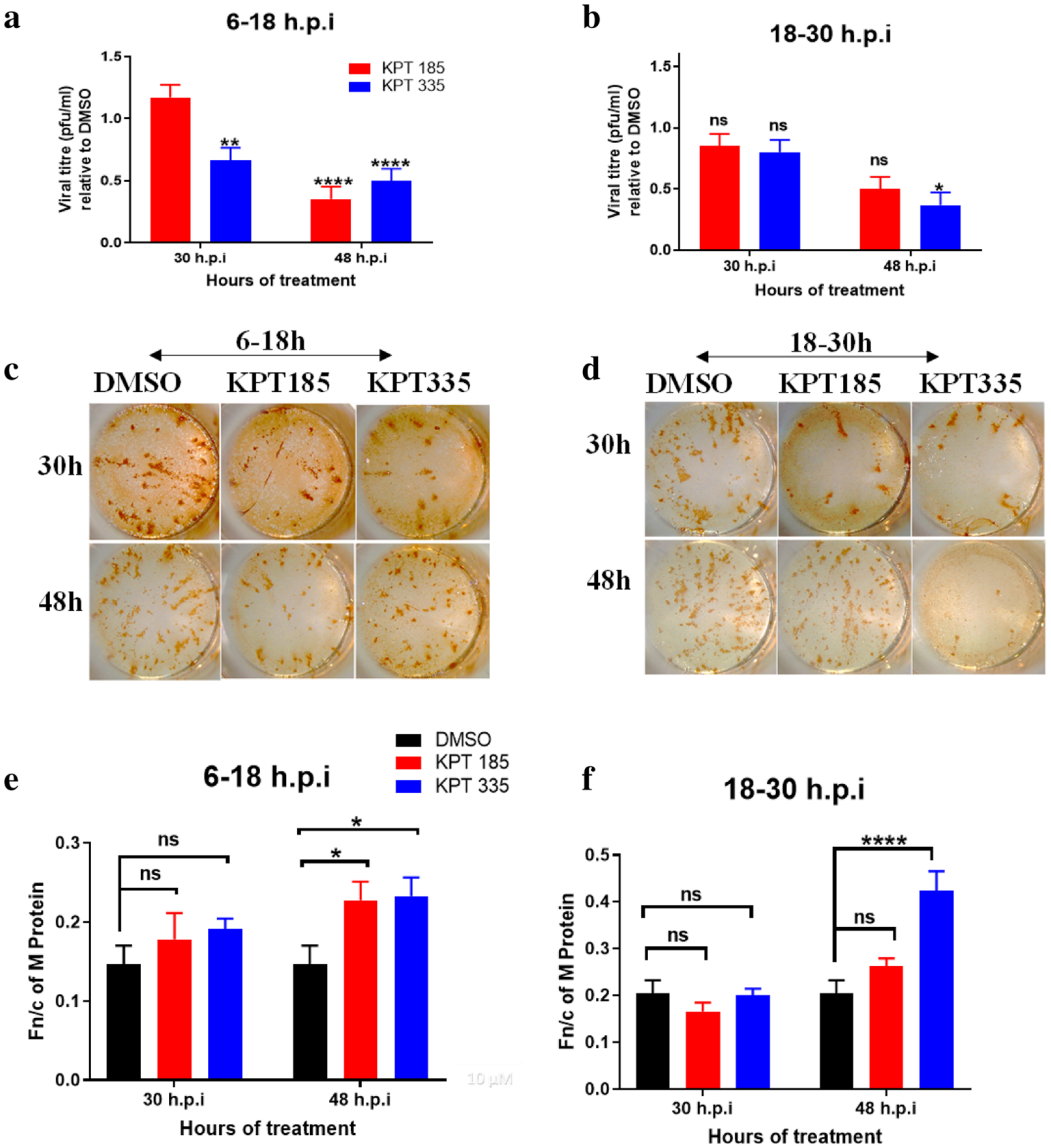
SINE compounds have a time-dependent effect against RSV replication and partially retain M protein to the nucleus. A549 cells were infected at MOI = 1 for 1 h followed by treatment with 1.5 μM of KPT-185 or KPT-335 or DMSO either from 6 to 18 h.p.i (early stage of infection) or 18 to 30 h.p.i (late stage). After the short-term treatment, the media was replaced with infection medium without the compounds and incubated up to 30 h.p.i or 48 h.p.i. The cell lysates were analysed using immunoplaque assays. Viral titre relative to DMSO-treated cell lysates after treatment with SINE compounds in the (**a**) early and (**b**) late stages of infection is shown. Please refer to Table S1 for individual virus titres. Representative images of plaques formed from cell lysates collected from infected cells treated during the (**c**) early and (**d**) late stages of infection. The same treatment was performed on cells grown overnight on coverslips in 12-well plates and fixed with 4% formaldehyde at 30 h.p.i or 48 h.p.i. Fixed cells were probed with mouse anti-M antibody and anti-mouse Alexafluor secondary antibody. Nuclear localization of M protein during the (**e**) early and (**f**) late stages of infection was determined as in Fig. 1. Data shown are mean ± SEM from three independent experiments. Statistical significance was determined using two-way ANOVA with Tukey’s post hoc test on GraphPad Prism v.8.4.3. ns: non-significant; *p < 0.05, ****p < 0.0001.

We have previously shown that short-term treatment with leptomycin B (LMB) disrupts XPO1 function in the early or late stages of RSV infection and leads to reduced viral replication^11^. To determine the efficacy of SINE compounds against RSV over the same time period, RSV-A2-infected A549 cells were treated with 1.5 µM of KPT-185 or KPT-335, from 6 to 18 h.p.i (early stage of infection), or 18–30 h.p.i (late stage of infection), and DMSO was used as the control. The SINE-containing medium was replaced with DMEM containing 2% FBS and 1 X PSN and incubated up to 30 h.p.i or 48 h.p.i. The viral titer in the lysate was determined using immunoplaque assay (Fig. 4).

Short-term treatment (12 h) with KPT-335 was more effective than KPT-185 in both early and late stages of RSV infection. Incubation up to 48 h.p.i (30 h in the absence of SINE compounds) reduced viral titre markedly compared to 12 h of treatment (18–30 h.p.i) (Fig. 4a,b). Relative to DMSO, treatment with KPT-335 in the early stages of infection significantly (p = 0.002) reduced viral titre. At 48 h.p.i, both KPT-335 and KPT-185 had significantly (p < 0.0001; to the same extent) reduced viral titre relative to DMSO. This correlates to higher efficacy of KPT-335 to reduce XPO1 expression and longer recovery period for the treated cells to restore XPO1 (Fig. 2). Treatment in the late stages of infection was effective at 48 h.p.i (p = 0.031), but not at 30 h.p.i (Fig. 4b,d). This suggests SINE compounds disrupt RSV replication after at least two rounds of replication. Reduced plaque formation is evident after short-term disruption (12 h) of XPO1 (Fig. 4a,b). This relates to the indirect reduction of RSV replication because of disruption of the XPO1 function and downstream processes.

The presence of fewer infectious virions in SINE-treated samples is shown in Fig. 4c,d. The number of plaques was substantially lower in SINE-treated A549 cells relative to DMSO-treated cells. Fewer comet-shaped plaques were observed in KPT-335-treated A549 cells compared to KPT-185-treated cells at both time points. Independent of time of addition, continuous treatment with the compounds up to 48 h.p.i showed increased efficacy against RSV relative to DMSO.

Immunofluorescence assays of A549 cells treated during the early and late stages of RSV infection were used to determine the subcellular localization of M protein. Congruent to decreased RSV replication, increased retention of RSV M protein was observed in SINE-treated A549 cells. Partial, yet statistically significant (p < 0.05), retention of M protein in the nucleus was observed following extended incubation up to 48 h.p.i regardless of period of treatment (Fig. 4e,f) as expected and shown previously for KPT-335. Both SINE compounds induced similar nuclear retention of M protein on early treatment up to 48 h.p.i (Fn/c = 0.227; p = 0.019 for KPT-185 and Fn/c = 0.232; p = 0.014 for KPT-335). Significantly (Fn/c = 0.424 and p < 0.0001) higher amount of M protein was localized to the nucleus on treatment with KPT-335 in the late stages of infection at 48 h.p.i relative to DMSO- or KPT-185-treated cells. This suggests increased accumulation of M protein in the nucleus with each round of replication and correlates with the pattern of reduction in viral titre on similar treatment (compare with Fig. 4b). Our data is in agreement with our previous study showing significant increase in nuclear localisation of M protein at 48 h.p.i. after treatment with KPT-335 early in infection^12^.

**Figure 5:**
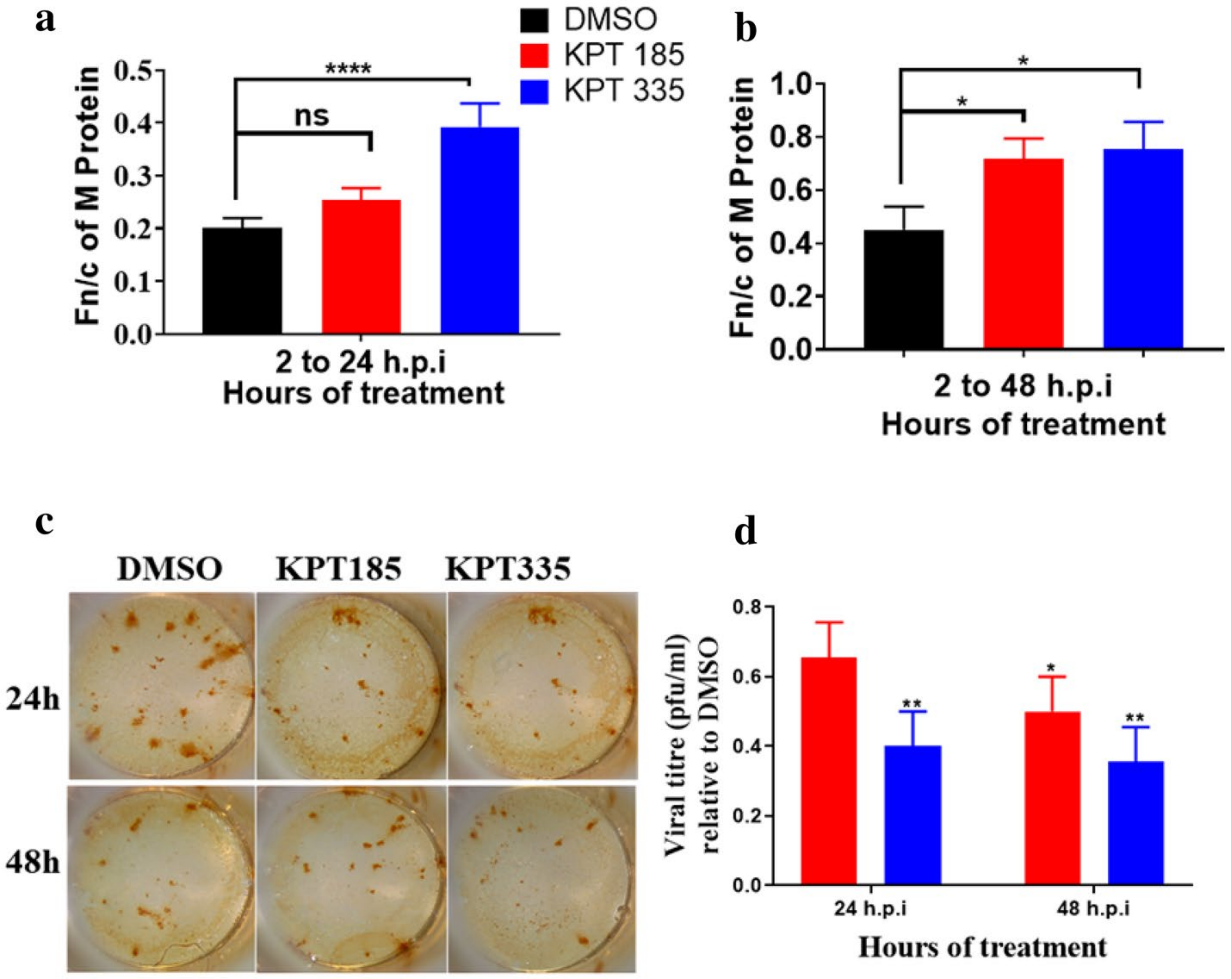
Therapeutic treatment with SINE compounds reduces RSV replication and disrupts export of M protein. A549 cells were infected at MOI = 1 for 1 h and treated from 2 h.p.i onwards with 1.5 μM of KPT-185 or KPT-335 or DMSO (control). The cell lysates were collected, or cells were fixed at 24 and 48 h.p.i. and analysed using immunofluorescence and immunoplaque assays. Nuclear localization of M protein in cells treated up to (**a**) 24 h.p.i and (**b**) 48 h.p.i. was determined as in Fig. 4. Data shown are mean ± SEM from three independent experiments. (**c**) Representative images of plaques formed from cell lysates collected from infected cells treated up to 24 and 48 h.p.i. (**d**) Viral titre relative to DMSO-treated cell lysates after treatment with SINE compounds. Data shown are mean ± SEM from quadruplicate samples from three independent experiments. Statistical significance was determined using two-way ANOVA with Tukey’s post hoc test on GraphPad Prism v.8.4.3. ns: non-significant; *p < 0.05, **p < 0.01, ****p < 0.0001.

Short-term SINE treatment (12 h) following RSV infection is not therapeutically germane and the effects of long-term SINE treatment were examined (Fig. 5). Following RSV-A2 infection, the cells were treated with SINE compounds or DMSO from 2 to 24 or 48 h.p.i. There was no significant difference in the amount of M retained in the nucleus between DMSO- or KPT-185-treated cells at 24 h.p.i (Fn/c = 0.254) (Fig. 5a). Further incubation up to 48 h.p.i showed a marginal increase in M protein being retained after treatment with KPT-185 (Fn/c = 0.715; p = 0.030) (Fig. 5b). KPT-335 effectively restricted export of M protein within 24 h.p.i (Fn/c = 0.392; p < 0.0001). At 48 h.p.i, there was an increase of protein retained to the nucleus in KPT-335-treated cells compared to levels at 24 h.p.i (Fn/c = 0.752; p = 0.032).

A pattern of reduction in viral titre similar to short-term treatment was observed with continuous SINE-treatment (Fig. 5c,d). 60% reduction in viral titre was observed by 24 h.p.i. with KPT-335 (p = 0.007 compared to DMSO), and at 48 h.p.i. following treatment with KPT-185 (p = 0.018 compared to DMSO) (Fig. 5d). Viral titre was significantly reduced following treatment with KPT-335 (compared to DMSO treatment) at both 24 h.p.i (p = 0.007) and 48 h.p.i. (p = 0.005). These findings suggest KPT-335 is more effective at disrupting XPO1-mediated export of M protein and retarding RSV replication in comparison to KPT-185 (also refer to Figs. 2, 3, 4).

**Figure 6:**
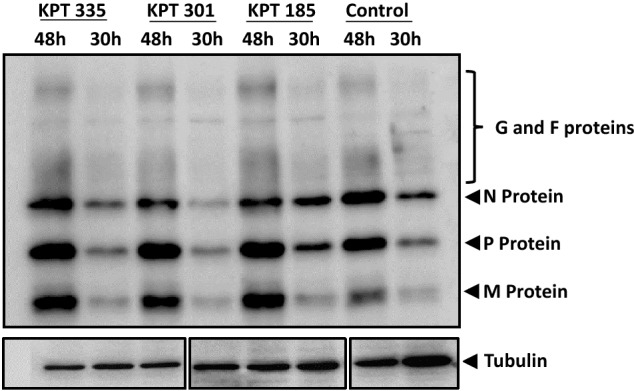
SINE compounds do not impact RSV protein expression. A549 cells were infected at MOI = 1 for 1 h and treated with 1.5 μM KPT-185, 1.5 μM KPT-335 or 50 μM KPT-301 from 2 to 30 h.p.i or to 48 h.p.i. 0.5 μM of DMSO was used as control. The cell lysates were analyzed by Western blotting. The blot was probed with goat α-RSV (1:1000) and rabbit α-Tubulin (1:1000), bound antibody was detected with horseradish peroxidase-conjugated secondary antibody (1:5000) and Enhanced Chemiluminescence (ECL, Perkin Elmer) kit. Images were taken using Li-Cor Odyssey Fc infrared imaging system and Image Studio Lite software. Tubulin was used as loading control. Image is representative of three independent experiments. Please refer to Fig. S2 for full length blots corresponding to the cropped blots presented here.

The absence of observable changes in RSV protein expression following treatment with SINE compounds suggests the inhibition of XPO1 does not affect RSV protein expression substantially (Fig. 6). Negligible changes in the level of RSV G, F, N, P or M proteins were detected in RSV-infected cells after treatment with SINE compounds up to 48 h.p.i compared to vehicle control.

Disruption of XPO1 is efficacious for inhibiting RSV replication following either prophylactic^12^ or therapeutic treatment with SINE compounds. This is most likely related to the inhibition of XPO1-mediated export and reduction of XPO1 in treated cells. The antiviral effect of SINE compounds could be a cumulative effect of reduced XPO1, disruption of XPO1-mediated export, partial nuclear retention of M, and impact on downstream sequences of other host pathways, including the cell cycle and expression of cytokines and chemokines.

### Treatment with SINE compounds delays cell cycle progression and affects expression of IL-8, IFN-λ and IFN-β

Both SINE compounds and RSV are known to impact the cell cycle. XPO1 mediates the nuclear exit of Cyclin Dependent Kinase (CDK)-cyclins such as cyclin B1 (which regulates transition from G2 to M phase), tumor suppressor proteins including p53, p21, Rb and FOXO that regulate CDK-cyclins and the progression of the cell cycle^28,29^. Previous studies with SINE compounds have shown reduced cell division in cancer cells^30^. RSV subverts the progression of cell cycle at both mRNA and protein levels.

**Figure 7:**
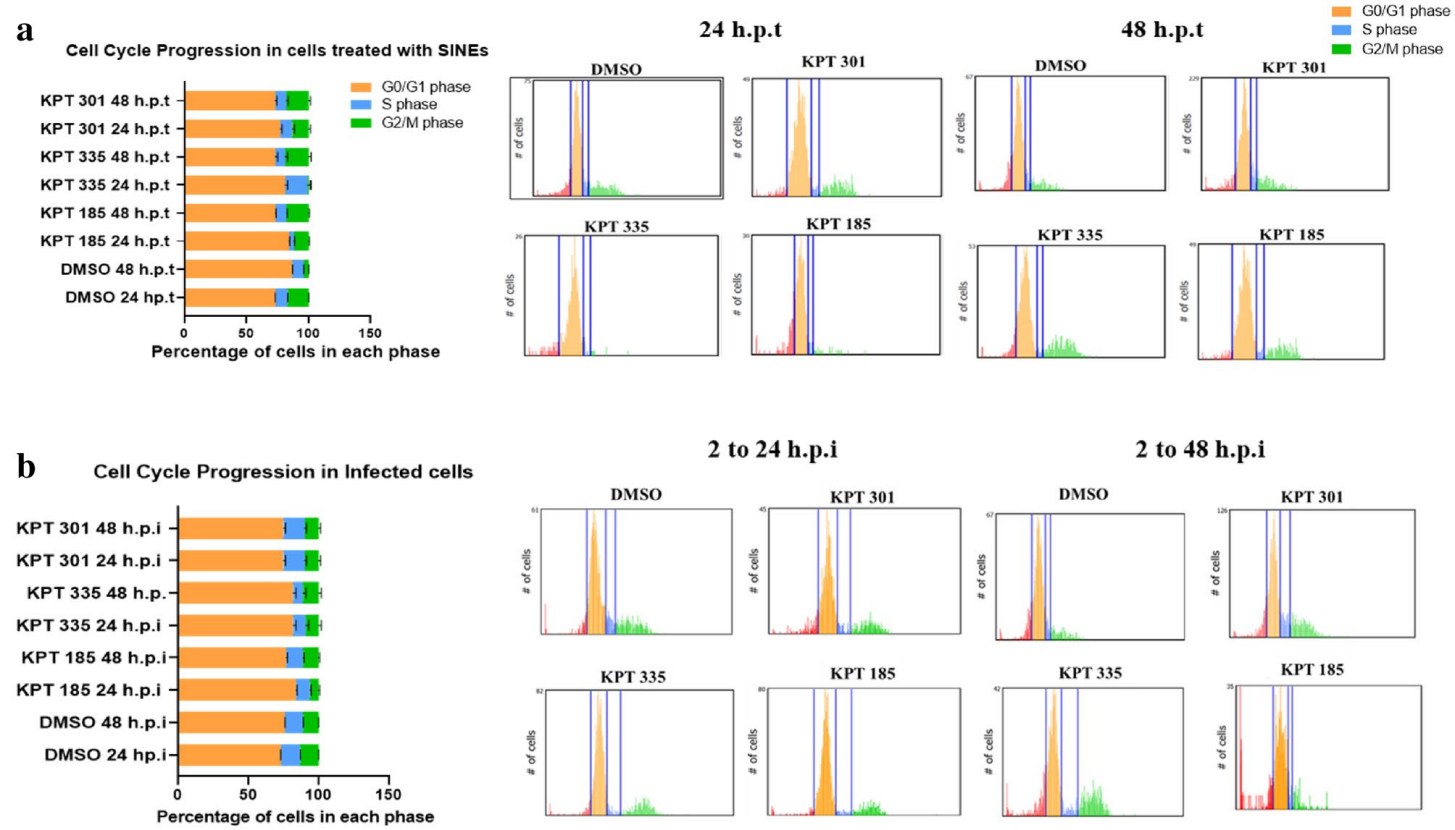
SINE compounds delay cell cycle progression. Sub-confluent monolayer of A549 cells were incubated for 24 h in Serum Free DMEM to allow synchronization of cell cycles to G0/G1 phase. (**a**) Non-infected cells were incubated for 24 h or 48 h in infection medium containing DMSO, 1.5 µM KPT-185 or 1.5 µM KPT-335. (**b**) Cells were infected with RSV-A2 (MOI = 1) for 1 h, further incubated for 2 h without the virus and treated with SINE compounds or DMSO for 24 or 48 h. The cells were trypsinized and fixed in 80% ice-cold ethanol. Tali™ Cell Cycle Kit and Tali^®^ Image-Based Cytometer were used to determine the percent of cells in each phase of the cell cycle. Bar charts on the left depict distribution of cells in each cell cycle phase; data shown are mean ± SEM. Each sample was tested in duplicate and data shown are from two independent experiments. The percentage of cells distributed in each phase of the cell cycle was plotted using GraphPad Prism v.8.4.3. Representative histograms for SINE- or DMSO-treated cells are shown on the right. The red region indicates cells in the G0 phase, yellow G1 phase, blue S phase and green G2/M phase.

In non-infected cells, the cell cycle distribution in DMSO-treated A549 cells at 24 h.p.i was 73% in the G0/G1 phase, 10% in the S phase and 17% in the G2/M phase, as expected^31,32^. Treatment with SINE compounds significantly altered the distribution of cells in each phase of the cell cycle (Fig. 7a). Treatment with KPT-185 reduced the percentage of cells in the S phase (84% in G0/G1, 4% in S and 12% in G2/M phase) within 24 h.p.i (p = 0.004 relative to DMSO) while KPT-335-treatment reduced the percentage of cells in the G2/M phase (p < 0.0001 relative to DMSO; 81% in G0/G1, 18% in S and 1% in G2/M) (Fig. 7a; representative histograms shown on the right). Treatment with KPT-301 had no effect on cell cycle distribution, which was similar to that of DMSO-treated cells (77% in G0/G1, 10% in S and 13% in G2/M). Previous studies in cancer cells have shown treatment with SINE compounds do not induce apoptosis as a consequence of XPO1 inhibition. Reduced expression of XPO1 may also disrupt the timely translocation of cell cycle regulators^58^. At 48 h.p.i, cell cycle distribution in SINE-treated cells was similar to DMSO-treated cells at 24 h.p.i (73% in G0/G1, 8% in S and 19% in G2/M phase). Taken together with our previous work^12^, this suggests treatment with KPT-185 or KPT-335 induces a delay in cell cycle progression.

At 24 h.p.i, majority of the RSV-infected A549 cells treated with DMSO were in G0/G1 (73%), 14% in S and 13% in the G2/M (Fig. 7b). For RSV-infected A549 cells treated with KPT-185, there was an increase in cells in G0/G1 phase (84%), followed by 10% in S, and 6% in G2/M. Similarly, treatment with KPT-335 increased the proportion of A549 cells in G0/G1 phase (82%), with the remaining cells equally distributed between the S and G2/M phases (9% each) (Fig. 7b). Our data suggests RSV infection resulted in some arrest in the S phase, while treatment with SINE compounds resulted in arrest in the G0/G1 phase.

**Figure 8:**
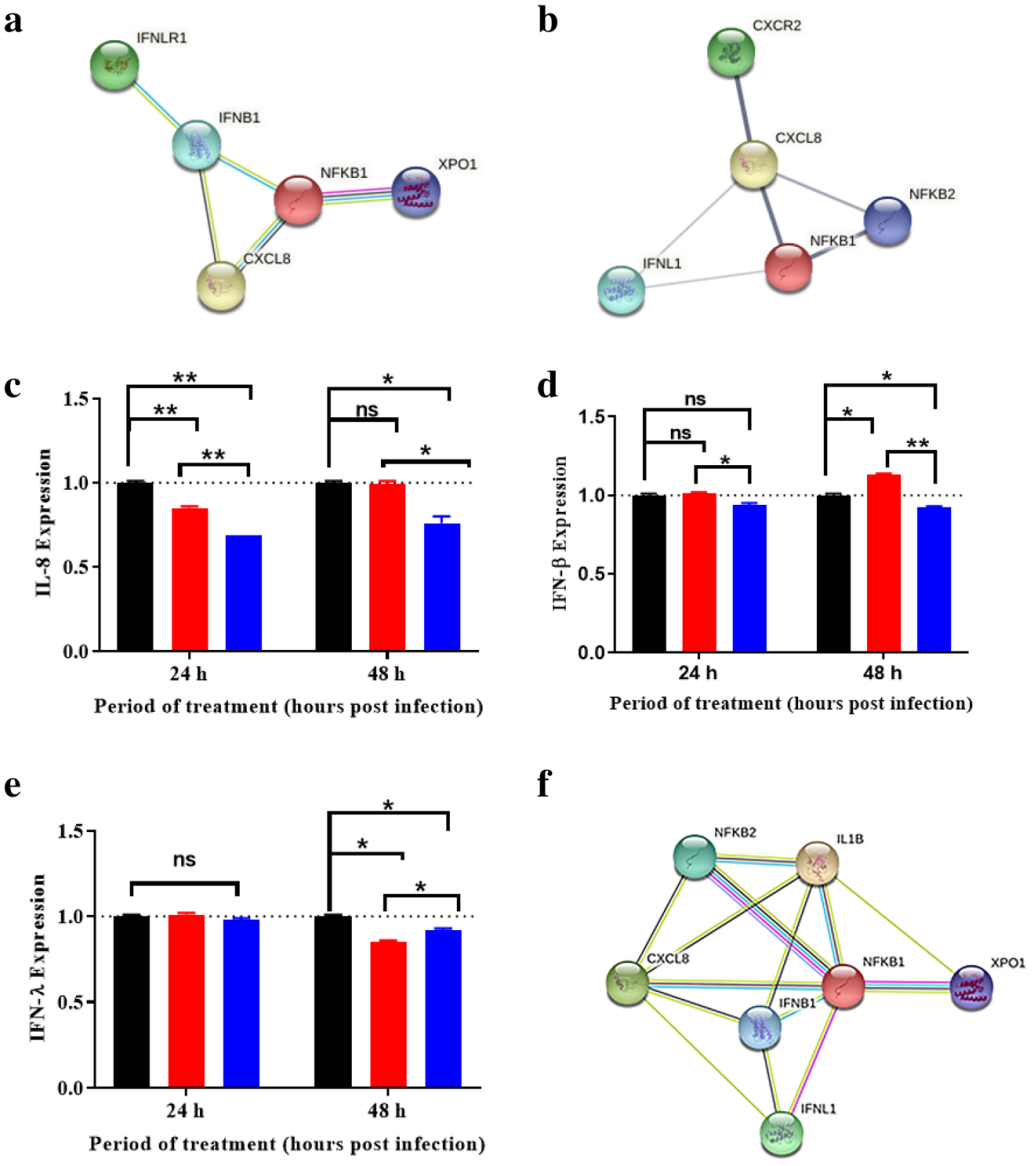
Treatment with SINE compounds reduces NFκB-mediated cytokine and interferon expression. Protein–protein interaction between IFN-λ, IFN-β, IL-8, NFκβ and XPO1 determined using STRING: functional protein association networks (https://string-db.org/). The summary of the interactions between the query proteins (*IFNLR1*- IFN-λ receptor 1; *IFNB1*- IFN-1β; *CXCL8*- IL-8; *CXCR2*- CXC chemokine receptor 2; *IFNL1*- IFN-λ1, *IL-1B*- IL-1β) was modified as shown. (**a**) NFKB1 is dependent on XPO1 to exit the nucleus and mediate the expression of IFNB1 and CXCL8. The latter goes on to activate the IFNLR1 receptor and induce an antiviral response. (**b**) Chemokine-dependent signalling between CXCR2 that activates IL-8, which in return is augmented by NFκβ (*NFKB1* or *NFKB2*) and IFN-λ1 (*IFNL1*). A549 cells were infected at MOI = 1 for 1 h and treated with 1.5 µM KPT-185 or KPT-335 or DMSO (control) from 2 to 24 or 48 h.p.i. Culture supernatants were collected and the amounts of (**c**) IL-8, (**d**) IFN-β and (**e**) IFN-λ were measured using ELISA kits. The expression of the cytokine and interferons shown are mean ± SEM relative to the control using GraphPad Prism v.8.4.3. Statistical significance was determined using 2-way ANOVA with Tukey’s post hoc test; ns: non-significant, *p < 0.05, **p < 0.01. Each experiment was performed in triplicate and data reported are from three independent experiments. (**f**) The expression of IL-8, IFN-β and IFN-λ are affected by interaction with NFκβ (*NFKB1* or *NFKB2*) and IL-1β (*IL1B*). Black arrows indicate direct interaction, dotted arrows indicate alternate interactions, blue arrows indicate interactions between cytokines and chemokines.

RSV induces a time-dependent increase in the expression of cytokines and chemokines that are regulated by NFκβ through the Rel/NFκB or p50/p65 pathway, which is actively involved in cell differentiation, host immune response and in the transcription of many inflammatory cytokines^33,34^. Analysis of protein interactions using the STRING database highlights the relationship between IL-8, IFN-β and IFN-λ expression and NFκβ-mediated signalling and the role of XPO1 therein (Fig. 8a,b)^35–37^. Chemokine expression is often mediated via NFκβ, JAK-STAT pathways or by activating protein 1 (AP1)-mediated transcription^38^. Exit of NFκB (p105) (*NFKB1* in Fig. 8a) via XPO1 pathway leads to increased expression of IFN-β (*IFNB1*) and IL-8 (*CXCL8*) (Fig. 8a). Both NFκB (*NFKB1* in Fig. 8b) and *CXCL8* can interact with IFN-λ1, a critical antiviral and immunomodulatory cytokine in epithelial cells^39,40^.

We have previously shown that treatment with KPT-335 up to 24 h.p.i. had no effect on IL-8 or IFNγ levels in RSV infected cells. We were interested to determine if XPO1 inhibition by SINE compounds would affect the expression of IL-8, IFN-β and IFN-λ after longer treatment. Previous studies have shown RSV replication induces a steady state of IL-8 production^41^. Relative to DMSO, treatment with KPT-335 significantly reduced IL-8 expression in infected A549 cells (p = 0.0010 at 24 h.p.i and p = 0.028 at 48 h.p.i; Fig. 8c). Treatment with KPT-185 marginally reduced IL-8 expression (p = 0.009) relative to DMSO at 24 h.p.i but was less effective compared to KPT-335 (Fig. 8c). No significant effect on IL-8 expression was observed after treatment with KPT-185 from 2 to 48 h.p.i relative to DMSO (Fig. 8c). The data suggests that treatment with KPT-335 induces an early and consistent decrease in IL-8 expression over 48 h relative to DMSO while KPT-185 has a short-term effect. Our data are somewhat in contradiction to our previous findings^12^ and may be due to the higher dose of KPT-335 used in this study (1.5 μM compared to 1 μM in the previous study).

RSV infection of primary airway epithelial cells in vitro and nasopharyngeal samples from infants infected with RSV results in dysregulated expression of IFNs including IFN-β and IFN-λ that provide antiviral resistance to host cells^42,43^. No significant effect on IFN-β expression was detected following treatment with SINE compounds at 24 h.p.i. relative to DMSO-treated cells (Fig. 8d). However, there was a significant difference in IFN-β expression between SINE treated cells, with a lower expression in KPT-335-treated cells relative to KPT-185 (p = 0.038; Fig. 8d). At 48 h.p.i, a marginal yet significant change was observed in SINE-treated cells relative to DMSO (p = 0.012 for KPT-185 and p = 0.030 for KPT-335; Fig. 8d). A marginal, yet significant, reduction in IFN- λ was also observed at 48 h.p.i relative to DMSO-treated cells (p = 0.0283 for KPT-185 and p = 0.030 for KPT-335), but not 24 h.p.i. (Fig. 8e).

The small effect of SINE treatment on IFN production suggests the involvement of alternate pathways affecting their expression^44^. For example, RSV induces the expression of IL-1β (IL-1B; Fig. 8f) which can increase the expression of IL-8 and IFN-β and in turn IFN-λ, bypassing the NFκβ-mediated signalling pathway^45,46^. Also, transient inhibition of XPO1 may have a short-lived effect which was evident in short-term treatment, but this temporary disruption may not be sufficient to have an overall impact on interferon production.

## Discussion

The therapeutic administration of KPT-185 and KPT-335 significantly reduced RSV replication in a dose- and time-dependent manner in cell culture, while KPT-301 was ineffective against RSV. Both KPT-335 and KPT-185 had low cytotoxicity, disrupted XPO1-mediated export, reversibly reduced the amount of XPO1 in treated cells and delayed cell cycle progression within 24 h of treatment. KPT-301 had low cytotoxicity but showed no inhibitory effects against RSV, did not reduce XPO1 levels and had no effect on cell cycle progression.

SINE compounds have been extensively characterized as chemotherapeutics for various solid and hematologic malignancies. Over-expression of XPO1 enables cancer cells to prematurely export Tumour Suppressor Proteins (TSPs) such as p53, p73, p21, p27, FOXO (1, 3a, and 4), PP2A, BRCA1 and BRCA2, and E2F4 and evade apoptosis and promote malignancy^14,23,47,48^. Inhibition or reduction of XPO1 expression forces the nuclear retention, accumulation, and functional activation of TSPs to limit oncogenesis^49–51^. The mechanism of action of SINE compounds is similar to LMB, binding to the Cys528 residue located within the Nuclear Export Signal (NES) groove of XPO1. Unlike LMB, SINE compounds are smaller, bind to the NES groove exclusively through hydrophobic interactions and are not hydrolyzed after conjugation. These attributes lead to the reversible nature of interaction and improved tolerance in non-malignant cells^52^. SINE compounds bind to XPO1 long enough to kill cancer cells, but their reversible nature allows them to be released in time to spare normal cells^52^.

Selective inhibition of XPO1-mediated transport is being actively pursued as a promising therapeutic target against viruses^8^, including SARS-CoV-2^53^. Inhibition of nuclear export of M protein by LMB-mediated disruption of XPO1 was shown to reduce RSV titres and site-directed mutation of the XPO1-binding domain in M protein completely inhibited viral replication^13^. This finding highlights the importance of XPO1 in the RSV lifecycle. Inhibition of XPO1-mediated transport was found effective both in vitro and in vivo against influenza A and B strains^17^. Treatment with SINE compounds was also effective against Venezuelan equine encephalitis virus (VEEV)^16^ and against opportunistic viruses that commonly affect immunocompromised patients such as Epstein-Barr virus, human cytomegalovirus, adenoviruses in vitro^18^. Recent studies have identified XPO1 as a key host protein modulated by the Nsp-4, -9 and Orf 6 proteins of SARS-CoV-2 and suggest that SINE compounds (ClinicalTrials.gov NCT04349098) could be potentially used for antiviral therapy^54,55^.

Therapeutic administration of KPT-185 or KPT-335 reduced XPO1 expression and partially retained M protein to the nucleus. Transient inhibition of XPO1 has a continued effect in reducing viral titre with successive replication cycles. Short-term treatment (12 h) with SINE compounds in either early or late stages of infection reduced RSV titres even after removing the compound. Continuous treatment with SINE compounds showed a similar reduction of RSV titres within 48 h of treatment. Reduced viral titers could translate to reduced virus spread and decreased lung involvement and disease pathogenesis*.*

In terms of cytotoxicity and disruption of the XPO1 function, KPT-335 was more effective than KPT-185 while KPT-301 was not effective. KPT-185 was the lead compound designed primarily for in vitro studies, and its orally bioavailable analog, KPT 251 has been successfully shown to have preclinical efficacy against various haematological and solid cancers in mice models^19^. KPT-335 was the first chemotherapeutic agent approved for treatment of canine lymphoma^23,56^. Both KPT-335 and KPT-185 were almost equally effective at disrupting XPO1-mediated export (1.5 µM). A high IC_50_ and poor disruption of XPO1 function rules out KPT-301 as a candidate against RSV despite having low cytotoxicity. This is in agreement with other studies that have evaluated its anti-cancer or antiviral activity including Jorquera et al.^12^, Lundberg et al.^16^; Perwitasari et al.^17^ and Widman et al.^18^.

Treatment with KPT-185 or KPT-335 induced a dose- and time-dependent nuclear accumulation of M protein but did not affect the total amount of the protein present in infected cells. The selective disruption of XPO1-mediated transport, shown using both pGFP-Rev (NES) transfected cells and nuclear localization of M protein in infected cells, is the primary mechanism of action of SINE compounds against viral replication. Disruption of XPO1 function results in partial M protein-nuclear accumulation and significant reduction in viral titre at 48 h.p.i., suggesting the disruption of M protein has an accumulative effect leading to reduced infectious virions with each replication cycle. Since M protein is conserved across RSV A and B strains and it exclusively traffics between subcellular compartments using XPO1^12,13^, it is probable that administration of KPT-185 and KPT-335 would potentially be effective against all RSV strains by subverting the nuclear export pathway used by the host cell.

In contrast to the marked reduction in viral protein expression following SINE-treatment against influenza, VEEV and opportunistic viruses affecting immunocompromised individuals^16–18^, transient inhibition of XPO1 during RSV replication did not detectably affect RSV protein production. This could be due to RSV protein synthesis and viral replication being localized to the cytoplasm as opposed to nuclear-replicating viruses^57^.

It is likely that in addition to partial nuclear accumulation of M protein, inhibition of XPO1 may alter other pathways that depend on nuclear export, including cell cycle progression and NFκβ-inflammatory pathways. Both pathways are subverted or manipulated during RSV infection and likely have a direct effect on RSV replication^58,59^. Treatment with SINE compounds could impact downstream pathways regulated by XPO1 and have been shown to cause cell cycle arrest, increase in inflammation and apoptosis in cancer cells^14,56,60–62^. RSV causes cell cycle arrest of the host cells in the S phase probably to promote its replication^63,64^. Treatment with SINE compounds in non-infected cells caused a delay in cell cycle progression, reducing the percentage of cells entering the S phase at 24 h.p.t followed by recovery by 48 h.p.t. This suggests the reversible interaction of KPT-185 or KPT-335 with XPO1 does not cause a permanent arrest of the cell cycle, rather a delay in progression, causing cells to remain longer in each phase. This data correlates with the longer recovery time for the XPO1 levels, increased nuclear accumulation and larger reduction in virus titre in KPT-335 treated cells.

RSV infection induces a strong inflammatory response within the airways^33,34^. This response is induced and stimulated primarily through the Rel/NFκB or p50/p65 pathway, which is actively involved in cell differentiation, host immune response and transcription of many inflammatory cytokines^33,34^. Continuous treatment with KPT-185 or KPT-335 significantly reduced IL-8 by 48 h.p.t. A marginal yet significant change in IFN-β and IFN-λ was also observed at 48 h.p.t. This may be beneficial in vivo since the inflammatory response would be reduced but the antiviral response to detect and eliminate viral particles might not be affected. The limited impact of SINE treatment on the inflammatory markers tested may be because NFκβ-mediated signalling is only one of the ways by which these markers are activated.

## Conclusion

In conclusion, KPT-185 and KPT-335 transiently reduce XPO1 expression, which recovers within 24 h of removal of the compound. The selective, reversible inhibition of XPO1 by therapeutic administration of KPT-185 or KPT-335 reduces RSV replication, probably with minimal effect in non-infected cells. Disruption of XPO1 function results in partial retention of RSV M protein in the nucleus, delays cell cycle progression and alters the induction of IL-8, IFN-β and -λ. Our current data adds to the growing evidence of XPO1 as an effective antiviral target against a broad range of viruses.

## Materials and methods

### Cells, RSV and SINE compounds

Human type II respiratory epithelial (A549) cells (ATCC CRL-185™) were grown in Dulbecco’s Modified Eagle Medium (DMEM) (Sigma, St. Louis, MO) supplemented with 10% (v/v) heat-inactivated Foetal Bovine Serum (FBS) (Bovogen, VIC, Australia) and 1× penicillin–streptomycin-neomycin (PSN; Sigma, VIC, Australia). Cells were maintained in a humidified 37 °C incubator supplied with 5% CO_2_. RSV-A2 (ATCC^®^ VR-1540) was grown in Vero cells (ATCC CCL-81) as described^12^. KPT-185, KPT-335, and KPT-301 (all from Karyopharm) were prepared in DMSO as 10 mM stocks and stored at − 20 °C until use. Final working concentrations of SINE compounds were prepared in DMEM supplemented with 2% (v/v) heat inactivated FBS and 1× PSN. KPT-185 is a potent XPO1 inhibitor in vitro but has reduced pharmacokinetics properties, making it unsuitable for in vivo use, and KPT-301 is its trans-isomer. KPT-185 and KPT-301 were used in this study as positive and negative controls, respectively. Equivalent concentration of DMSO (Sigma) was used as control.

### Transfection

Transfection of A549 cells was performed using Lipofectamine 2000 (Life Technologies, VIC, Australia) using a 1:1 mix of DNA and reagent. The cells were transfected with pGFP (pGFP-DESTC) or pGFP-Rev (NES) (pEPI-GFP-REV (2-116)) constructs^13^. SINE compounds or DMSO were added to A549 cells 18 h post-transfection. The cells were examined at 6 h post treatment (h.p.t) using confocal laser scanning microscopy (CLSM) and analyzed as described below.

### Lactate Dehydrogenase (LDH) Assay

CytoTox96^®^ non-radioactive cytotoxicity assay (Promega, Sydney, Australia) was used to determine cytotoxicity following the manufacturer’s instructions. Briefly, overnight cultures of A549 cells were treated with increasing concentration of SINE compounds prepared in DMEM containing 2% FBS and 1× PSN. 48 h.p.t, the supernatant was incubated with LDH reagent (Promega) at room temperature for 30 min in the dark, followed by addition of stop solution (Promega) and absorbance was measured at 490 nm. The average optical density (OD) of untreated cells was subtracted from each experimental well. The mean OD values of lysed cells were considered 100% cytotoxic and used to calculate the percent cytotoxicity of SINE compounds. The percent cytotoxicity versus the log10 concentration of SINE compounds was plotted using GraphPad Prism v.8.4.3 and the values were fitted to a non-linear regression curve to determine the 50% cytotoxic concentration (CC_50_).

### Plaque reduction assay

Overnight cultures of A549 cells were infected with RSV-A2 at multiplicity of infection (MOI) = 1 and incubated for 1 h with periodic gentle swirling. The viral inoculum was decanted and replaced with DMEM supplemented with 2% (v/v) heat inactivated FBS and 1× PSN. After 2 h of incubation, the infected cells were treated with increasing doses of SINE compounds up to 48 h.p.i. Cells treated with DMSO alone were used as control. At the end of incubation, the supernatant was decanted, and the cells were fixed with methanol + 2% H_2_O_2_ followed by air-drying overnight. The viral titre was determined using an immuno-plaque assay with a goat anti-RSV antibody (Merck Millipore, VIC, Australia) and donkey anti-goat IgG-HRP (Thermofisher, VIC, Australia). The percentage reduction in viral titre associated with SINE treatment was determined in comparison to DMSO-treated cells. The values were fitted to a non-linear regression curve using GraphPad Prism v.8.4.3 to determine the IC_50_, the concentration of drug required to induce 50% inhibition of viral replication.

### Immuno-plaque assay

The fixed cells were blocked at room temperature for 30 min in 1% BSA (Sigma) prepared in 1× PBS followed by incubation in 1:1000 dilution of goat anti-RSV antibody (Merck Millipore) in 1× PBS for 2 h at room temperature. The plate was washed four times with 1× PBS and dry blotted, followed by incubation with 1:1000 dilution of donkey anti-goat-IgG-HRP antibody (Abcam, VIC, Australia) in 1× PBS for 2 h at room temperature in the dark. The plate was washed as before and incubated with freshly prepared SigmaFast™ Diaminobenzidine (DAB) Peroxidase Substrate (dissolved in Tris Buffer Saline (pH 7.4) supplemented with 6.67 μl of 30% H_2_O_2_ (Sigma) per 10 mg DAB for 2–6 h at room temperature in the dark. Images of the plaques were taken using Leica EZ24W stereomicroscope and Leica Application Suite software. The plaque forming units (PFU/ml) = (average number of plaques per well)/(Viral volume × dilution factor). The viral titre in the treated samples relative to DMSO-treated samples was plotted using GraphPad Prism v.8.4.3. Statistical significance was determined using two-way ANOVA and Tukey’s post hoc test.

### Immunofluorescence assay

A549 cells were grown overnight on glass coverslips and infected at MOI = 0.5 or 1 with RSV-A2. The cells were treated with SINE compounds for the specified time interval. Cells treated with DMSO alone were used as controls. At the indicated times p.i., cells were fixed with 4% formaldehyde, permeabilized with 0.1% Triton X-100, and immune-stained with a monoclonal antibody specific for RSV M protein (MAbαM)^65^ and Alexa Fluor 488-conjugated secondary antibody (Life Technologies). Hoechst 33342 (ThermoFisher) diluted in 1× PBS was used for nuclear staining. The coverslips were mounted on slides using Fluorescence mounting medium (Dako) and analysed using CLSM.

### CLSM and image analysis

Fixed or live A549 cells were imaged as described previously^13^. Briefly, digitized fluorescent cell images were collected using a Nikon Ti Eclipse confocal laser-scanning microscope (CLSM) with a Nikon 60 × /1.40 oil immersion lens (Plan Apo VC OFN25 DIC N2; optical section of 0.5 μm) and the NIS Elements AR software. Data from four individual scans were averaged to obtain the final images. Images were analysed as previously described^11,66^ using Fiji ImageJ (vr. 1.52 s). The fluorescence intensity above background (Fb) in the nucleus (Fn) compared to that in the cytoplasm (Fc) was used to determine the nuclear to cytoplasmic fluorescence ratio (Fn/c). The Fn/c values were plotted using GraphPad Prism v.8.4.3.

### Analysis of protein expression

A549 cells were infected for 1 h with RSV-A2 (MOI = 1) or left uninfected, and treated with SINE compounds or DMSO from 2 . to 24 or 48 h.p.i. Cells were lysed in freshly prepared RIPA buffer (150 mM NaCl, 1.0% Triton X-100, 0.5% sodium deoxycholate, 10% SDS, 50 mM Tris, pH 8.0; protease inhibitor cocktail, and PhosSTOP inhibitor tablet; (all from Sigma)). The supernatant was collected after centrifugation at 13,000×*g*, at 4 °C for 30 min, and analysed using Western blotting.

The lysates were mixed with 6× Laemmli sample buffer, boiled, and electrophoresed on a 12% polyacrylamide gel; 10 µl of each sample was loaded per lane. The separated proteins were transferred to nitrocellulose and probed with goat anti-RSV antibody, mouse anti-XPO1 (BD Biosciences, NSW, Australia) or mouse anti-α/β-tubulin (Genesearch, QLD, Australia) monoclonal antibodies diluted 1:1000 in 1% skim milk in 1× PBS (pH 7.2) containing 0.1% Tween 20 (PBST). Bound antibody was detected with horseradish peroxidase-conjugated secondary antibodies diluted 1:5000 in 1% skim milk in PBST. The bound antibody was detected using Enhanced Chemiluminescence (ECL, Perkin Elmer) and imaged on the LiCor Odyssey^®^ Fc Imaging System with Image Studio™ Lite software. Wherever required, blots were stripped using stripping buffer (2% SDS, 62.5 mM Tris–HCl (pH 6.8) and 114.4 mM β-mercaptoethanol (all from Sigma)) at 50 °C for 10 min, followed by washing in PBST, blocking in 4% skim milk in 1× PBST and re-probed with primary antibodies overnight as required. Intensity of the bands was measured using Fiji ImageJ (vr. 1.52s). Values were expressed as arbitrary units relative to total protein present (for RSV) or to corresponding intensity of tubulin (for XPO1), used as the loading control.

### Cell cycle analysis

A549 cells in 6-well plates were serum starved for 24 h in serum-free DMEM (Sigma). These synchronized cells were infected with RSV-A2 at MOI = 0.5 for 1 h, as described previously or left uninfected. At 2 h.p.i, cells were treated with 1.5 μM of KPT-185, KPT-335 or DMSO for 24 or 48 h. Cells were trypsinized, fixed in ice-cold 80% ethanol, stained using Tali™ Cell Cycle Kit as per the manufacturer’s recommendations and analysed using Tali^®^ Image-Based Cytometer. The threshold gate for each cell cycle phase was set on the Tali^®^ Image-Based Cytometer during the analysis of each sample. The percentage of cells in each phase of the cell cycle under different conditions was estimated. The average percentage of cells in each phase of the cell cycle and standard error were plotted using GraphPad Prism v.8.4.3. Significance was determined using two-way ANOVA and Tukey’s post hoc test.

### Cytokine and interferon expression in infected cells treated with SINE compounds

A549 cells infected (MOI = 0.5) with RSV-A2 for 1 h were treated with 1.5 μM KPT-185 or KPT-335 or DMSO from 2 to 24 or 48 h.p.i. Culture supernatants were collected and clarified of cellular debris by centrifugation. Interleukin (IL)-8 (R&D Systems), Interferon (IFN)-λ (R&D Systems) and IFN-β (ELISAkit.com) expression was determined by ELISA as per the manufacturer’s recommendations. Fold-change in expression in SINE-treated cells was calculated relative to DMSO-treated cells. Significance was determined using two-way ANOVA and Tukey’s post hoc test on GraphPad Prism v.8.4.3.

### Protein–protein interactions between NFκβ, IFNs, IL-8 and XPO1

Protein–protein interactions based on known functional associations were determined using STRING: functional protein association networks (https://string-db.org/). The online database depicts a protein network of genome-wide functional connectivity. The relevant proteins were searched under “Multiple Proteins”, and “*Homo sapiens*”. The query proteins, i.e. NFkB1, NFkB2, XPO1, IL-8, IFN-λ and IFN-β were selected. The result shows a summary of the interactions between the query proteins.

## Supplementary Information

Supplementary Information.
