# Supplementary material for: Reversible disruption of XPO1-mediated nuclear export inhibits respiratory syncytial virus (RSV) replication
Source: Sci Rep. 2021 Sep 28;11:19223. doi: 10.1038/s41598-021-98767-2 (PMC8479129; doi:10.1038/s41598-021-98767-2)
Supplement: Supplementary file 1 — Supplementary Information. [file 41598_2021_98767_MOESM1_ESM.pptx]

## Slide 1
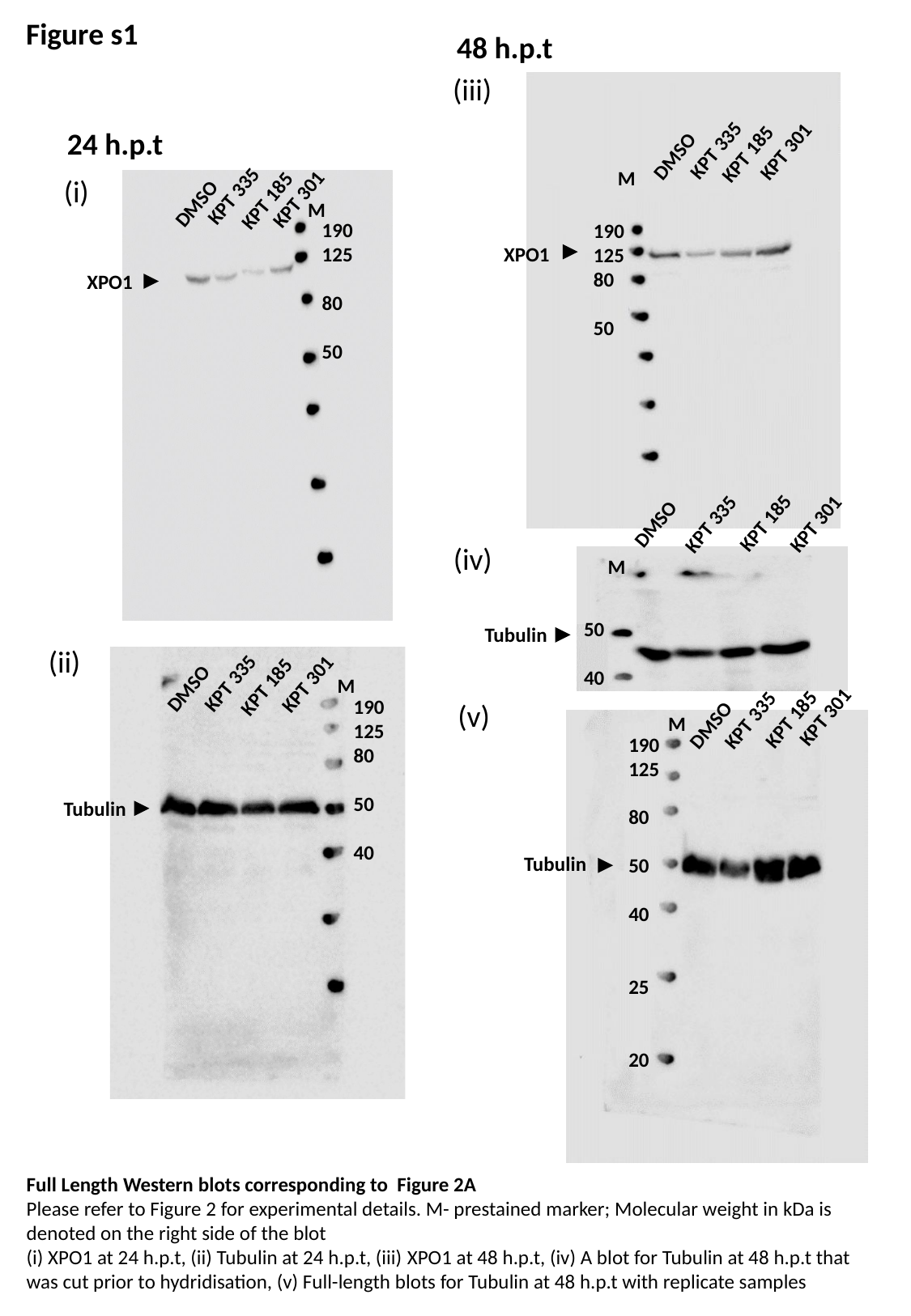

Figure s1
48 h.p.t
(iii)
24 h.p.t
KPT 335
KPT 301
KPT 185
DMSO
M
(i)
KPT 335
KPT 301
KPT 185
DMSO
M
190
125
80
50
190
125
80
50
XPO1
XPO1
KPT 185
KPT 301
KPT 335
DMSO
(iv)
M
50
40
Tubulin
(ii)
KPT 335
KPT 301
M
KPT 185
DMSO
190
125
80
50
40
(v)
KPT 301
KPT 185
KPT 335
M
DMSO
190
125
80
50
40
25
20
Tubulin
Tubulin
Full Length Western blots corresponding to Figure 2A
Please refer to Figure 2 for experimental details. M- prestained marker; Molecular weight in kDa is denoted on the right side of the blot
(i) XPO1 at 24 h.p.t, (ii) Tubulin at 24 h.p.t, (iii) XPO1 at 48 h.p.t, (iv) A blot for Tubulin at 48 h.p.t that was cut prior to hydridisation, (v) Full-length blots for Tubulin at 48 h.p.t with replicate samples

## Slide 2
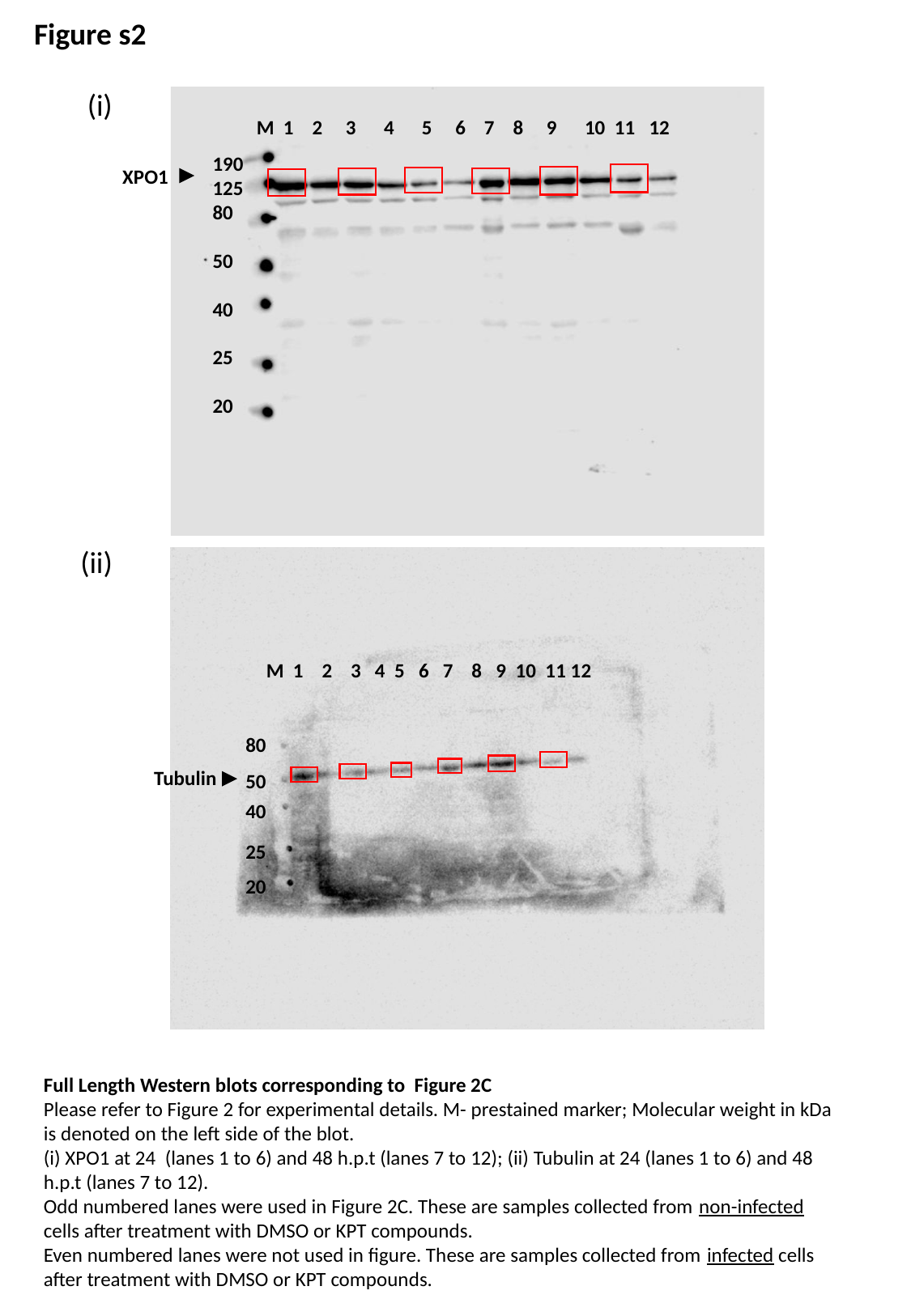

Figure s2
(i)
 M 1 2 3 4 5 6 7 8 9 10 11 12
190
125
80
50
40
25
20
XPO1
(ii)
 M 1 2 3 4 5 6 7 8 9 10 11 12
80
50
40
25
20
Tubulin
Full Length Western blots corresponding to Figure 2C
Please refer to Figure 2 for experimental details. M- prestained marker; Molecular weight in kDa is denoted on the left side of the blot.
(i) XPO1 at 24 (lanes 1 to 6) and 48 h.p.t (lanes 7 to 12); (ii) Tubulin at 24 (lanes 1 to 6) and 48 h.p.t (lanes 7 to 12).
Odd numbered lanes were used in Figure 2C. These are samples collected from non-infected cells after treatment with DMSO or KPT compounds.
Even numbered lanes were not used in figure. These are samples collected from infected cells after treatment with DMSO or KPT compounds.

## Slide 3
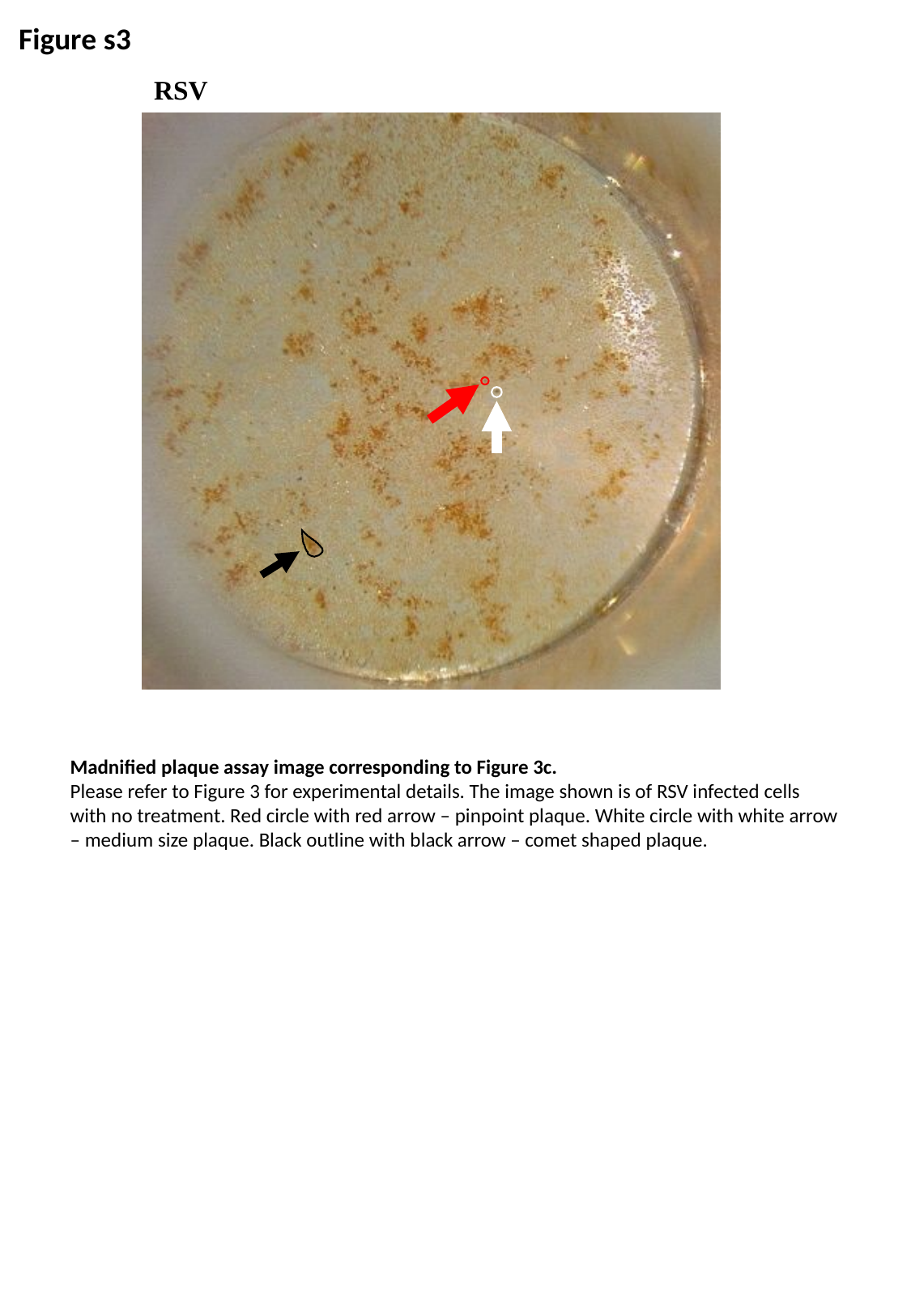

Figure s3
RSV
Madnified plaque assay image corresponding to Figure 3c.
Please refer to Figure 3 for experimental details. The image shown is of RSV infected cells with no treatment. Red circle with red arrow – pinpoint plaque. White circle with white arrow – medium size plaque. Black outline with black arrow – comet shaped plaque.

## Slide 4
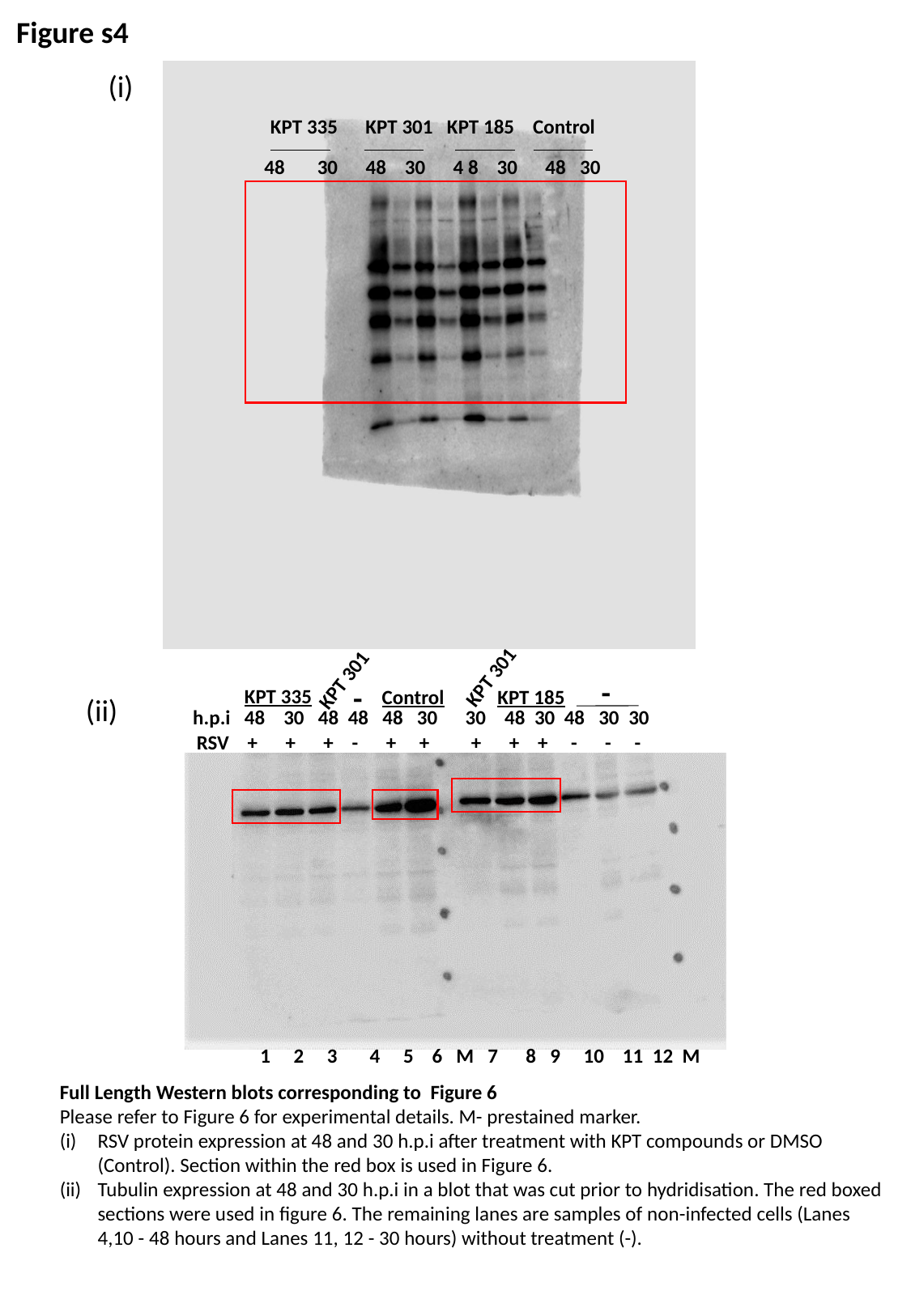

Figure s4
(i)
KPT 335 KPT 301 KPT 185 Control
48 30 48 30 4 8 30 48 30
KPT 301
KPT 301
-
-
KPT 335
Control
KPT 185
(ii)
h.p.i 48 30 48 48 48 30 30 48 30 48 30 30
RSV + + + - + + + + + - - -
 1 2 3 4 5 6 M 7 8 9 10 11 12 M
Full Length Western blots corresponding to Figure 6
Please refer to Figure 6 for experimental details. M- prestained marker.
RSV protein expression at 48 and 30 h.p.i after treatment with KPT compounds or DMSO (Control). Section within the red box is used in Figure 6.
Tubulin expression at 48 and 30 h.p.i in a blot that was cut prior to hydridisation. The red boxed sections were used in figure 6. The remaining lanes are samples of non-infected cells (Lanes 4,10 - 48 hours and Lanes 11, 12 - 30 hours) without treatment (-).

## Slide 5
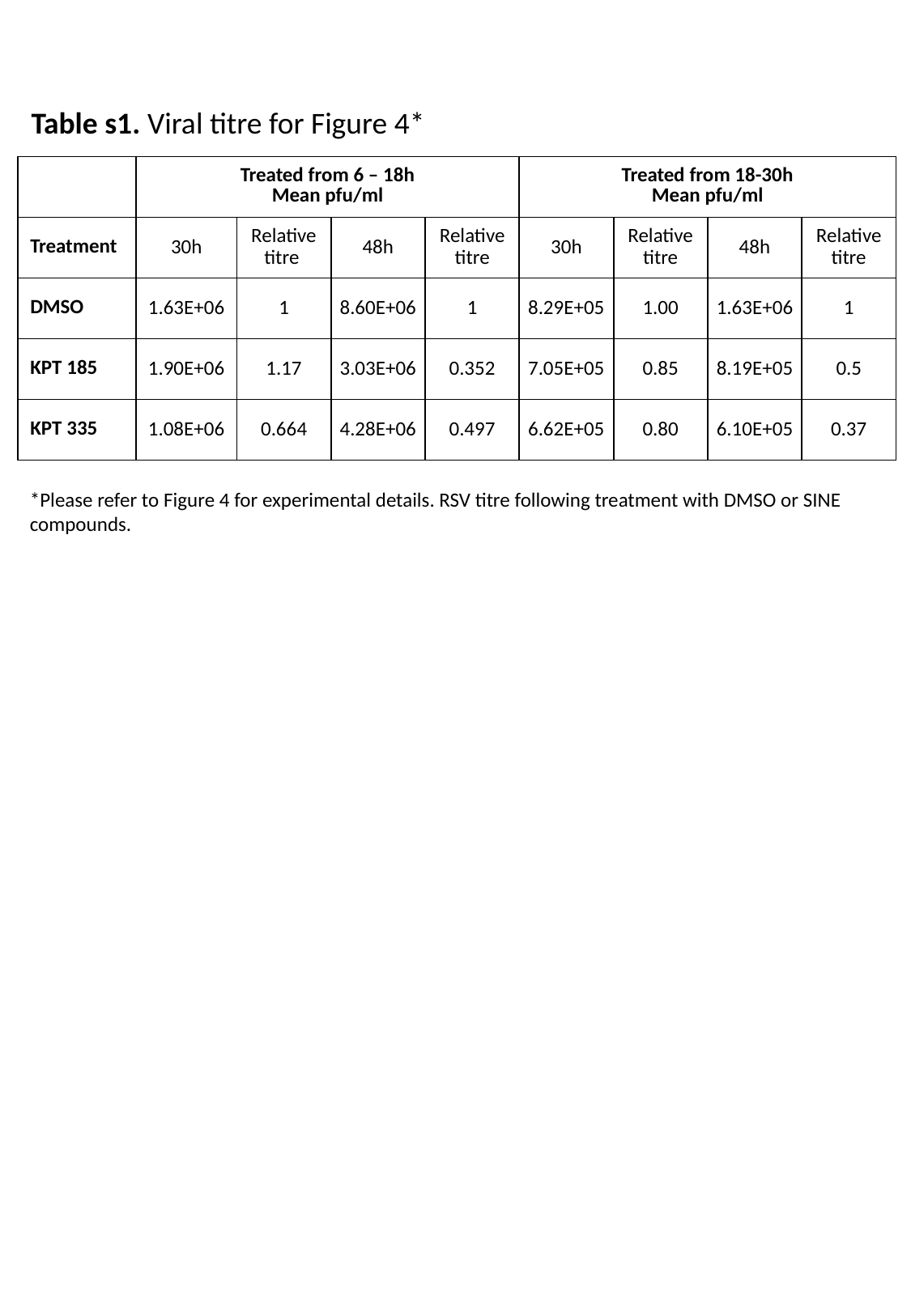

Table s1. Viral titre for Figure 4*
| | Treated from 6 – 18h Mean pfu/ml | | | | Treated from 18-30h Mean pfu/ml | | | |
| --- | --- | --- | --- | --- | --- | --- | --- | --- |
| Treatment | 30h | Relative titre | 48h | Relative titre | 30h | Relative titre | 48h | Relative titre |
| DMSO | 1.63E+06 | 1 | 8.60E+06 | 1 | 8.29E+05 | 1.00 | 1.63E+06 | 1 |
| KPT 185 | 1.90E+06 | 1.17 | 3.03E+06 | 0.352 | 7.05E+05 | 0.85 | 8.19E+05 | 0.5 |
| KPT 335 | 1.08E+06 | 0.664 | 4.28E+06 | 0.497 | 6.62E+05 | 0.80 | 6.10E+05 | 0.37 |
*Please refer to Figure 4 for experimental details. RSV titre following treatment with DMSO or SINE compounds.
